# Comparative efficacy and acceptability of 21 antidepressant drugs for the acute treatment of adults with major depressive disorder: a systematic review and network meta-analysis

**DOI:** 10.1016/S0140-6736(17)32802-7

**Published:** 2018-04-07

**Authors:** Andrea Cipriani, Toshi A Furukawa, Georgia Salanti, Anna Chaimani, Lauren Z Atkinson, Yusuke Ogawa, Stefan Leucht, Henricus G Ruhe, Erick H Turner, Julian P T Higgins, Matthias Egger, Nozomi Takeshima, Yu Hayasaka, Hissei Imai, Kiyomi Shinohara, Aran Tajika, John P A Ioannidis, John R Geddes

**Affiliations:** a Department of Psychiatry, University of Oxford, Oxford, UK; b Oxford Health NHS Foundation Trust, Warneford Hospital, Oxford, UK; c Oxford Centre for Human Brain Activity, Department of Psychiatry, Warneford Hospital, Oxford, UK; d Department of Health Promotion and Human Behavior, Kyoto University Graduate School of Medicine and School of Public Health, Kyoto, Japan; e Institute of Social and Preventive Medicine, University of Bern, Bern, Switzerland; f School of Medicine, Paris Descartes University, Paris, France; g INSERM, UMR1153 Epidemiology and Statistics, Sorbonne Paris Cité Research Center, METHODS Team, Paris, France; h Cochrane France, Paris, France; i Department of Psychiatry and Psychotherapy, Technische Universität München, Munich, Germany; j Department of Psychiatry, Radboud University Nijmegen Medical Centre, Nijmegen, Netherlands; k Behavioral Health and Neurosciences Division, VA Portland Health Care System, Portland, OR, USA; l Department of Psychiatry and Department Pharmacology, Oregon Health & Science University, Portland, OR, USA; m School of Social and Community Medicine, University of Bristol, Bristol, UK; n Department of Medicine, Department of Health Research and Policy, Department of Biomedical Data Science, and Department of Statistics, Stanford University, Stanford, CA, USA; o Meta-Research Innovation Center at Stanford, Stanford University, Stanford, CA, USA

## Abstract

**Background:**

Major depressive disorder is one of the most common, burdensome, and costly psychiatric disorders worldwide in adults. Pharmacological and non-pharmacological treatments are available; however, because of inadequate resources, antidepressants are used more frequently than psychological interventions. Prescription of these agents should be informed by the best available evidence. Therefore, we aimed to update and expand our previous work to compare and rank antidepressants for the acute treatment of adults with unipolar major depressive disorder.

**Methods:**

We did a systematic review and network meta-analysis. We searched Cochrane Central Register of Controlled Trials, CINAHL, Embase, LILACS database, MEDLINE, MEDLINE In-Process, PsycINFO, the websites of regulatory agencies, and international registers for published and unpublished, double-blind, randomised controlled trials from their inception to Jan 8, 2016. We included placebo-controlled and head-to-head trials of 21 antidepressants used for the acute treatment of adults (≥18 years old and of both sexes) with major depressive disorder diagnosed according to standard operationalised criteria. We excluded quasi-randomised trials and trials that were incomplete or included 20% or more of participants with bipolar disorder, psychotic depression, or treatment-resistant depression; or patients with a serious concomitant medical illness. We extracted data following a predefined hierarchy. In network meta-analysis, we used group-level data. We assessed the studies' risk of bias in accordance to the Cochrane Handbook for Systematic Reviews of Interventions, and certainty of evidence using the Grading of Recommendations Assessment, Development and Evaluation framework. Primary outcomes were efficacy (response rate) and acceptability (treatment discontinuations due to any cause). We estimated summary odds ratios (ORs) using pairwise and network meta-analysis with random effects. This study is registered with PROSPERO, number CRD42012002291.

**Findings:**

We identified 28 552 citations and of these included 522 trials comprising 116 477 participants. In terms of efficacy, all antidepressants were more effective than placebo, with ORs ranging between 2·13 (95% credible interval [CrI] 1·89–2·41) for amitriptyline and 1·37 (1·16–1·63) for reboxetine. For acceptability, only agomelatine (OR 0·84, 95% CrI 0·72–0·97) and fluoxetine (0·88, 0·80–0·96) were associated with fewer dropouts than placebo, whereas clomipramine was worse than placebo (1·30, 1·01–1·68). When all trials were considered, differences in ORs between antidepressants ranged from 1·15 to 1·55 for efficacy and from 0·64 to 0·83 for acceptability, with wide CrIs on most of the comparative analyses. In head-to-head studies, agomelatine, amitriptyline, escitalopram, mirtazapine, paroxetine, venlafaxine, and vortioxetine were more effective than other antidepressants (range of ORs 1·19–1·96), whereas fluoxetine, fluvoxamine, reboxetine, and trazodone were the least efficacious drugs (0·51–0·84). For acceptability, agomelatine, citalopram, escitalopram, fluoxetine, sertraline, and vortioxetine were more tolerable than other antidepressants (range of ORs 0·43–0·77), whereas amitriptyline, clomipramine, duloxetine, fluvoxamine, reboxetine, trazodone, and venlafaxine had the highest dropout rates (1·30–2·32). 46 (9%) of 522 trials were rated as high risk of bias, 380 (73%) trials as moderate, and 96 (18%) as low; and the certainty of evidence was moderate to very low.

**Interpretation:**

All antidepressants were more efficacious than placebo in adults with major depressive disorder. Smaller differences between active drugs were found when placebo-controlled trials were included in the analysis, whereas there was more variability in efficacy and acceptability in head-to-head trials. These results should serve evidence-based practice and inform patients, physicians, guideline developers, and policy makers on the relative merits of the different antidepressants.

**Funding:**

National Institute for Health Research Oxford Health Biomedical Research Centre and the Japan Society for the Promotion of Science.

**Research in context**

**Evidence before this study**

Antidepressants are routinely used worldwide for the treatment of major depressive disorder, which is one of the most important global health challenges; however, in the scientific literature, there remains considerable debate about both their effectiveness as a group, and the potential differences in effectiveness and tolerability between individual drugs. With the marketing of new antidepressants and increasing numbers of trials published every year, an updated systematic review and network meta-analysis was required to synthesise the evidence in this important clinical area.

**Added value of this study**

This network meta-analysis represents a major update and extension of our previous study, which addressed 12 antidepressants with data for head-to-head comparisons only, and provides the best currently available evidence base to guide the choice about pharmacological treatment for adults with acute major depressive disorder. We now include a more comprehensive list of 21 antidepressants and placebo, consider three new clinical outcome measures and many potential effect modifiers, and use the most advanced statistical methodology for network meta-analysis to date.

**Implications of all the available evidence**

Our findings should inform clinical guidelines and assist the shared decision making process between patients, carers, and clinicians in routine practice on selecting the most appropriate antidepressant for adults with acute major depressive disorder. Future research should seek to extend network meta-analysis to combine aggregate and individual-patient data from trials in a so-called individual-patient data network meta-analysis. This analysis will allow the prediction of personalised clinical outcomes, such as early response or specific side-effects, and the estimate of comparative efficacy at multiple timepoints.

## Introduction

Psychiatric disorders account for 22·8% of the global burden of diseases.^1^ The leading cause of this disability is depression, which has substantially increased since 1990, largely driven by population growth and ageing.^2^ With an estimated 350 million people affected globally, the economic burden of depressive disorders in the USA alone has been estimated to be more than US$210 billion, with approximately 45% attributable to direct costs, 5% to suicide-related costs, and 50% to workplace costs.^3^ This trend poses a substantial challenge for health systems in both developed and developing countries, with the need to treat patients, optimise resources, and improve overall health care in mental health.

Grouped into various classes of drugs with slightly different mechanisms of action, antidepressants are widely used treatments for major depressive disorder, which are available worldwide. However, there is a long-lasting debate and concern about their efficacy and effectiveness, because short-term benefits are, on average, modest; and because long-term balance of benefits and harms is often understudied.^4^ Therefore, innovation in psychopharmacology is of crucial importance, but the identification of new molecular targets is difficult, primarily because of the paucity of knowledge about how antidepressants work.^5^ In routine practice, clinicians have a wide choice of individual drugs and they need good evidence to make the best choice for each individual patient. Network meta-analyses of existing datasets make it possible to estimate comparative efficacy, summarise and interpret the wider picture of the evidence base, and to understand the relative merits of the multiple interventions.^6^ Therefore, in this study, we aimed to do a systematic review and network meta-analysis to inform clinical practice by comparing different antidepressants for the acute treatment of adults with unipolar major depressive disorder.

## Methods

### Search strategy and selection criteria

We did a systematic review and network meta-analysis. We searched the Cochrane Central Register of Controlled Trials, CINAHL, Embase, LILACS database, MEDLINE, MEDLINE In-Process, PsycINFO, AMED, the UK National Research Register, and PSYNDEX from the date of their inception to Jan 8, 2016, with no language restrictions. We used the search terms “depress*” OR “dysthymi*” OR “adjustment disorder*” OR “mood disorder*” OR “affective disorder” OR “affective symptoms” combined with a list of all included antidepressants.

We included double-blind, randomised controlled trials (RCTs) comparing antidepressants with placebo or another active antidepressant as oral monotherapy for the acute treatment of adults (≥18 years old and of both sexes) with a primary diagnosis of major depressive disorder according to standard operationalised diagnostic criteria (Feighner criteria, Research Diagnostic Criteria, DSM-III, DSM-III-R, DSM-IV, DSM-5, and ICD-10). We considered only double-blind trials because we included placebo in the network meta-analysis, and because this study design increases methodological rigour by minimising performance and ascertainment biases.^7^ Additionally, we included all second-generation antidepressants approved by the regulatory agencies in the USA, Europe, or Japan: agomelatine, bupropion, citalopram, desvenlafaxine, duloxetine, escitalopram, fluoxetine, fluvoxamine, levomilnacipran, milnacipran, mirtazapine, paroxetine, reboxetine, sertraline, venlafaxine, vilazodone, and vortioxetine. To inform clinical practice globally, we selected the two tricyclics (amitriptyline and clomipramine) included in the WHO Model List of Essential Medicines). We also included trazodone and nefazodone, because of their distinct effect and tolerability profiles. Additionally, we included trials that allowed rescue medications so long as they were equally provided among the randomised groups. We included data only for drugs within the therapeutic range (appendix pp 133, 134). Finally, we excluded quasi-randomised trials and trials that were incomplete or included 20% or more of participants with bipolar disorder, psychotic depression, or treatment-resistant depression; or patients with a serious concomitant medical illness.

The electronic database searches were supplemented with manual searches for published, unpublished, and ongoing RCTs in international trial registers, websites of drug approval agencies, and key scientific journals in the field.^8^ For example, we searched ClinicalTrials.gov using the search term “major depressive disorder” combined with a list of all included antidepressants. We contacted all the pharmaceutical companies marketing antidepressants and asked for supplemental unpublished information about both premarketing and post-marketing studies, with a specific focus on second-generation antidepressants. We also contacted study authors and drug manufacturers to supplement incomplete reports of the original papers or provide data for unpublished studies.

Six pairs of investigators (ACi, TAF, LZA, SL, HGR, YO, NT, YH, EHT, HI, KS, and AT) independently selected the studies, reviewed the main reports and supplementary materials, extracted the relevant information from the included trials, and assessed the risk of bias. Any discrepancies were resolved by consensus and arbitration by a panel of investigators within the review team (ACi, TAF, LZA, EHT, and JRG).

The full protocol of this network meta-analysis has been published.^8^

### Outcomes

Our primary outcomes were efficacy (response rate measured by the total number of patients who had a reduction of ≥50% of the total score on a standardised observer-rating scale for depression) and acceptability (treatment discontinuation measured by the proportion of patients who withdrew for any reason).^8^ All-cause discontinuation was used as a measure for the acceptability of treatments, because it encompasses efficacy and tolerability.^9^ Secondary outcomes were endpoint depression score, remission rate, and the proportion of patients who dropped out early because of adverse events. When depressive symptoms had been measured with more than one standardised rating scale, we used a predefined hierarchy, based on psychometric properties and consistency of use across included trials.^8^ In the absence of information or supplemental data from the authors, response rate was calculated according to a validated imputation method.^10^ We recorded the outcomes as close to 8 weeks as possible for all analyses.^9^ If information at 8 weeks was not available, we used data ranging between 4 and 12 weeks (we gave preference to the timepoint closest to 8 weeks; if equidistant, we took the longer outcome). We checked trial protocols where available and compared published with unpublished data. We extracted data following a predefined hierarchy described in our protocol and gave priority to unpublished information in case of disagreement.^8^

### Data analysis

For studies published more than once (ie, duplicates), we included only the report with the most informative and complete data. Full details of the applied statistical approaches are provided in the protocol.^8^ We estimated summary odds ratios (ORs) for dichotomous outcomes and standardised mean differences (SMD, Cohen's *d*) for continuous outcomes using pairwise and network meta-analysis. In network meta-analysis, we used group-level data; the binomial likelihood was used for dichotomous outcomes and the normal likelihood for continuous outcomes. The study effect sizes were then synthesised using a random-effects network meta-analysis model. We accounted for the correlations induced by multi-group studies by using multivariate distributions. The variance in the random-effects distribution (heterogeneity variance) was considered to measure the extent of across-study and within-comparison variability on treatment effects. Additionally, in network meta-analysis, we assumed that the amount of heterogeneity was the same for all treatment comparisons. To assess the amount of heterogeneity, we compared the posterior distribution of the estimated heterogeneity variance with its predictive distribution.^11^ To rank the treatments for each outcome, we used the surface under the cumulative ranking curve (SUCRA) and the mean ranks.^12^ The transitivity assumption underlying network meta-analysis was evaluated by comparing the distribution of clinical and methodological variables that could act as effect modifiers across treatment comparisons.^8^ We did a statistical evaluation of consistency (ie, the agreement between direct and indirect evidence) using the design-by-treatment test^13^ and by separating direct evidence from indirect evidence.^14^

We assessed the studies' risk of bias in accordance to the Cochrane Handbook for Systematic Reviews of Interventions. Additionally, we assessed the certainty of evidence contributing to network estimates of the main outcomes with the Grading of Recommendations Assessment, Development and Evaluation (GRADE) framework.^15^

We evaluated whether treatment effects for the two primary outcomes were robust in subgroup analyses and network meta-regression using study year, sponsorship, depressive severity at baseline, dosing schedule, study precision (ie, small study effect), and novelty effect.^16^ The appendix (pp 133–36) summarises the definition of covariates. The sensitivity of our conclusions was evaluated by analysing the dataset with the following restrictions: studies with reported response rate, studies using accepted doses in all groups, studies with unpublished data, multi-centre studies, and head-to-head studies. We used comparison-adjusted funnel plots to investigate whether results in imprecise trials differ from those in more precise trials.^17^

We fitted all models in OpenBUGS (version 3.2.2)^18^ using the binomial likelihood for dichotomous outcomes, uninformative prior distributions for the treatment effects, and a minimally informative prior distribution for the common heterogeneity SD. We assumed uninformative priors—ie, N(0,1000)—for all meta-regression coefficients. Convergence of models was ensured by visual inspection of three chains and after considering the Brooks–Gelman–Rubin diagnostic. The codes of analyses, statistical details of the meta-analysis, and meta-regression models are presented in the appendix (pp 182, 183). Statistical evaluation of inconsistency and production of network graphs and result figures were done using the network and network graphs packages in Stata (version 14.2).^19^ Network meta-analyses of the primary outcomes were duplicated using the netmeta 0.9-6 package in R (version 3.4.0).^20^ The appendix (p 289) lists the changes to the original protocol. The study was done from March 12, 2012, to June 4, 2016, and data analysis was done from June 5, 2016, to Sept 18, 2017.

This study is registered with PROSPERO, number CRD42012002291.

### Data sharing

With the publication of this Article, the full dataset will be freely available online in Mendeley Data, a secure online repository for research data, which allows archiving of any file type and assigns a permanent and unique digital object identifier (DOI) so that the files can be easily referenced (DOI:10.17632/83rthbp8ys.2).

### Role of the funding source

The funder of this study had no role in study design, data collection, data analysis, data interpretation, writing of the report, or in the decision to submit for publication. ACi, TAF, GS, ACh, LZA, and YO had full access to all the data, and ACi was responsible for the decision to submit for publication.

## Results

**Figure 1:**
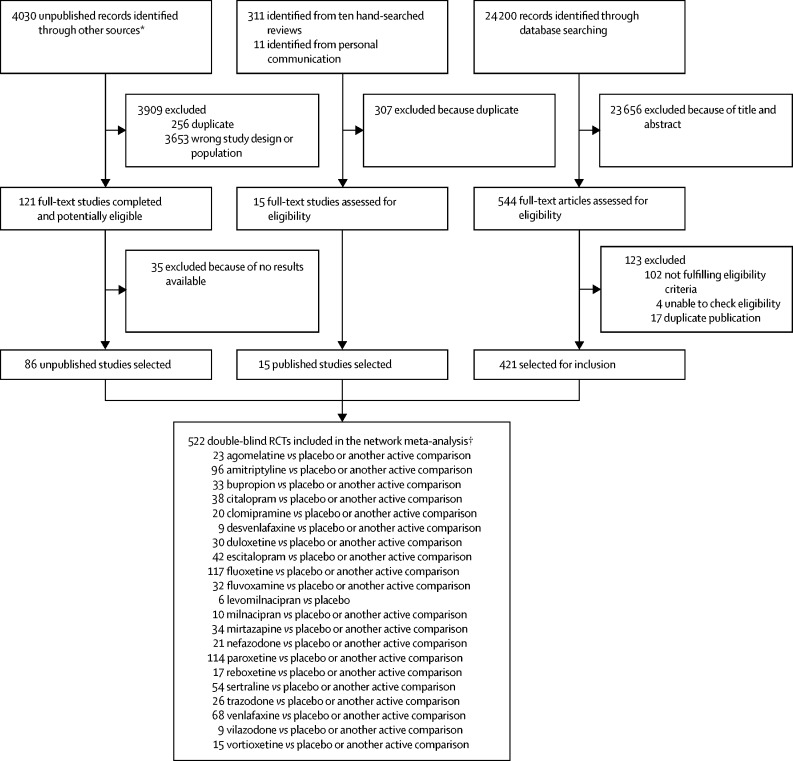
Study selection process RCTs=randomised controlled trials. *Industry websites, contact with authors, and trial registries. The total number of unpublished records is the total number of results for each drug and on each unpublished database source. †522 RCTs corresponded to 814 treatment groups.

28 552 citations were identified by the search and 680 potentially eligible articles were retrieved in full text (figure 1). We included 421 trials from the database search, 86 unpublished studies from trial registries and pharmaceutical company websites, and 15 from personal communication or hand-searching other review articles. Overall, 522 double-blind, parallel, RCTs (comprising 116 477 patients) done between 1979 and 2016, and comparing 21 antidepressants or placebo were included in the analysis (appendix pp 6–64). The appendix (pp 65–114) summarises the characteristics of included studies. The mean study sample size was 224 participants (SD 186). In total, 87 052 participants were randomly assigned to an active drug and 29 425 were randomly assigned to placebo. The mean age was 44 years (SD 9) for both men and women; 38 404 (62·3%) of 61 681 of the sample population were women. The median duration of the acute treatment was 8 weeks (IQR 6–8). 243 (47%) of 522 studies randomly assigned participants to three or more groups, and 304 (58%) of 522 were placebo-controlled trials. 391 (83%) of 472 were multi-centre studies and 335 (77%) of 437 studies recruited outpatients only. 252 (48%) of 522 trials recruited patients from North America, 37 (7%) from Asia, and 140 (27%) from Europe (59 [11%] trials were cross-continental and the remaining 34 [7%] were either from other regions or did not specify). The great majority of patients had moderate-to-severe major depressive disorder, with a mean reported baseline severity score on the Hamilton Depression Rating Scale 17-item of 25·7 (SD 3·97) among 464 (89%) of 522 studies. Response rate was imputed in 20 608 (17·7%) of 116 447 cases. Rescue medications (typically benzodiazepines or other sedative hypnotics) were allowed in 187 (36%) of 522 studies. 409 (78%) of 522 studies were funded by pharmaceutical companies. We retrieved unpublished information for 274 (52%) of the included trials. Consistent with the study protocol, the primary analysis was based on the 474 studies (comprising 106 966 patients) that used drugs within the licensed dose range (ie, the dosage approved by the regulatory agencies in the USA and Europe; appendix pp 133, 134).

**Figure 2:**
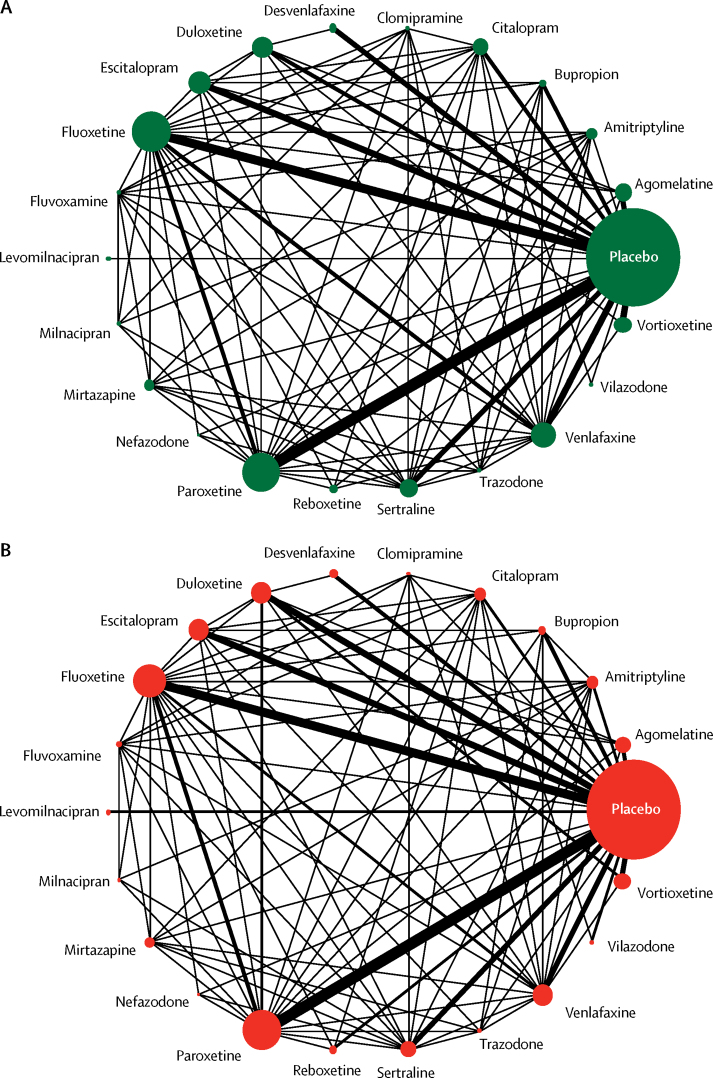
Network meta-analysis of eligible comparisons for efficacy (A) and acceptability (B) Width of the lines is proportional to the number of trials comparing every pair of treatments. Size of every circle is proportional to the number of randomly assigned participants (ie, sample size).

**Figure 3:**
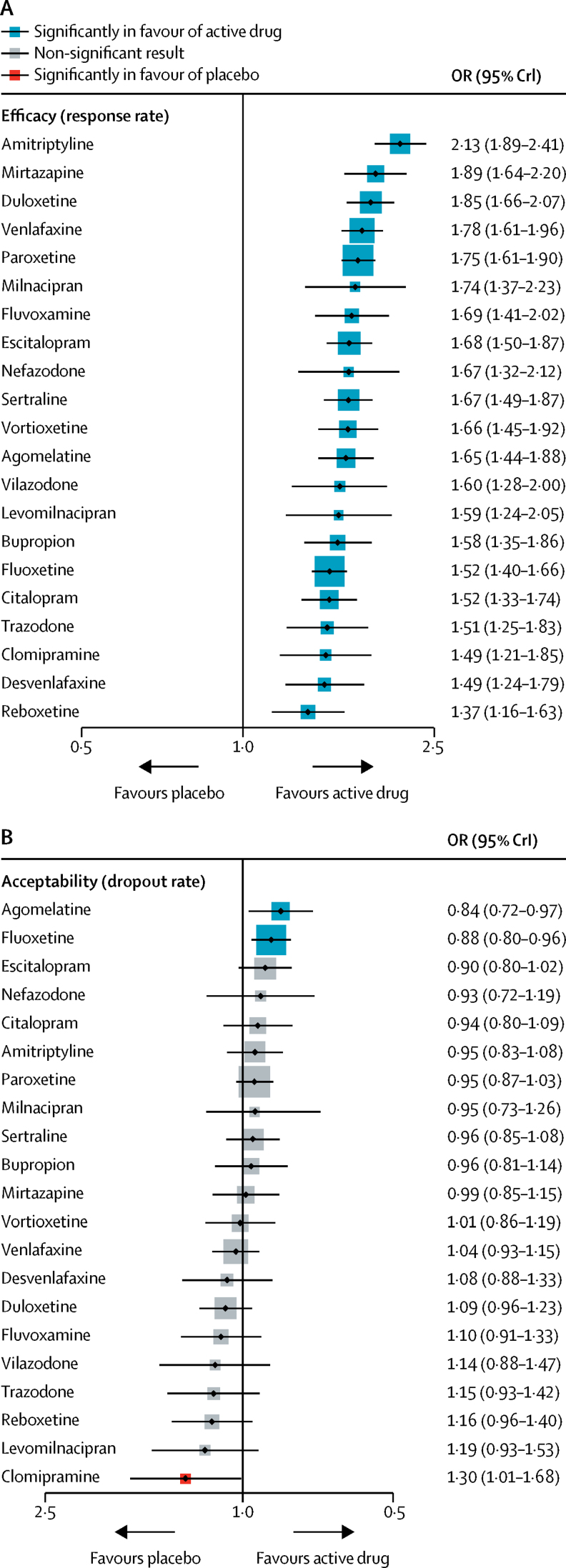
Forest plots of network meta-analysis of all trials for efficacy (A) and acceptability (B) Antidepressants were compared with placebo, which was the reference compound. OR=odds ratio. CrI=credible interval.

Figure 2 shows the network of eligible comparisons for efficacy and acceptability. All antidepressant drugs, except milnacipran, had at least one placebo-controlled trial. Only levomilnacipran was not directly compared with at least another active drug in any of the networks. The appendix (pp 139–44) provides detailed results of pairwise meta-analyses. Figure 3 shows the network meta-analysis' results for the primary outcomes. In terms of efficacy (432 RCTs, comprising 102 443 patients), all antidepressants were more effective than placebo, with ORs ranging between 2·13 (95% credible interval [CrI] 1·89–2·41) for amitriptyline and 1·37 (1·16–1·63) for reboxetine. In terms of acceptability (422 RCTs, comprising 99 787 patients), agomelatine (OR 0·84, 95% CrI 0·72–0·97) and fluoxetine (0·88, 0·80–0·96) were associated with fewer dropouts than placebo; by contrast, clomipramine was worse than placebo (1·30, 1·01–1·68).

The relative efficacy of antidepressants compared with placebo is also shown for remission (appendix pp 152, 153). The random-effects summary SMD for all antidepressants was 0·30 (95% CrI 0·26–0·34; p<0·0001; appendix pp 150, 151). In terms of dropouts due to adverse events, all active drugs were associated with higher withdrawal rates than placebo with ORs ranging between 1·64 and 4·44, and 95% CrI excluding the null, except agomelatine (OR 1·21, 95% CrI 0·94–1·56; appendix pp 154–55). For the full results of the secondary outcomes see the appendix (pp 150–55).

In the analysis of response rate, 8% of the loops were inconsistent (17 of 219 loops; p value of the design by treatment test was 0·063), and also 8% of the loops were inconsistent for dropouts (16 of 210 loops; p=0·219). The median heterogeneity variances were estimated at 0·044 (95% CrI 0·028–0·063) for response and 0·040 (0·023–0·062) for dropout, suggesting moderate-to-low heterogeneity. Subgroup meta-regression analyses revealed that the use of placebo in trials was the strongest explanation of heterogeneity and inconsistency in those evaluated. Exclusion of placebo-controlled trials resulted in a 24% relative reduction in heterogeneity variance for response and 45% for dropout. Additionally, we found that smaller and older studies presented larger effects of the active interventions versus placebo (in particular for amitriptyline, bupropion, fluoxetine, and reboxetine; appendix pp 182–96). The year of randomisation or study precision did not materially impact on the relative treatment effects between active interventions (appendix p 228). Overall, 46 (9%) of 522 trials were rated as high risk of bias, 380 (73%) trials as moderate, and 96 (18%) as low (appendix pp 115–32).

**Figure 4:**
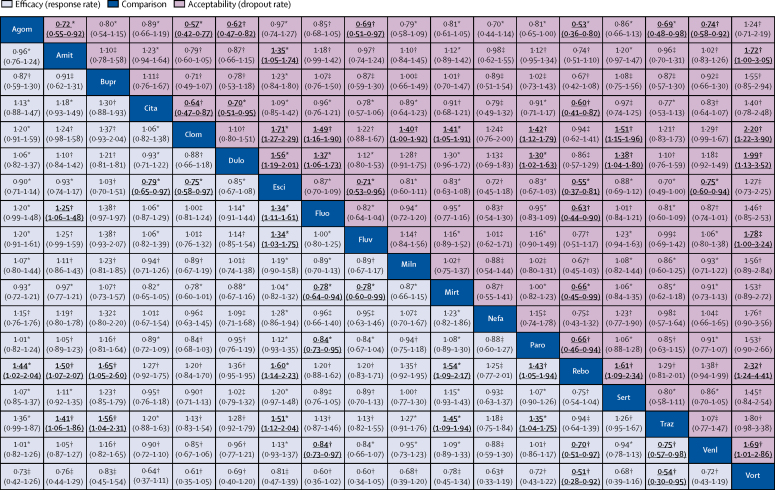
Head-to-head comparisons for efficacy and acceptability of the 21 antidepressants Drugs are reported in alphabetical order. Data are ORs (95% CrI) in the column-defining treatment compared with the row-defining treatment. For efficacy, ORs higher than 1 favour the column-defining treatment (ie, the first in alphabetical order). For acceptability, ORs lower than 1 favour the first drug in alphabetical order. To obtain ORs for comparisons in the opposite direction, reciprocals should be taken. Significant results are in bold and underscored. The certainty of the evidence (according to GRADE) was incorporated in this figure (appendix pp 231–65). OR=odds ratio. CrI=credible interval. Agom=agomelatine. Amit=amitriptyline. Bupr=bupropion. Cita=citalopram. Clom=clomipramine. Dulo=duloxetine. Esci=escitalopram. Fluo=fluoxetine. Fluv=fluvoxamine. Miln=milnacipran. Mirt=mirtazapine. Nefa=nefazodone. Paro=paroxetine. Rebo=reboxetine. Sert=sertraline. Traz=trazodone. Venl=venlafaxine. Vort=vortioxetine. *Moderate quality of evidence. †Low quality of evidence. ‡Very low quality of evidence.

**Figure 5:**
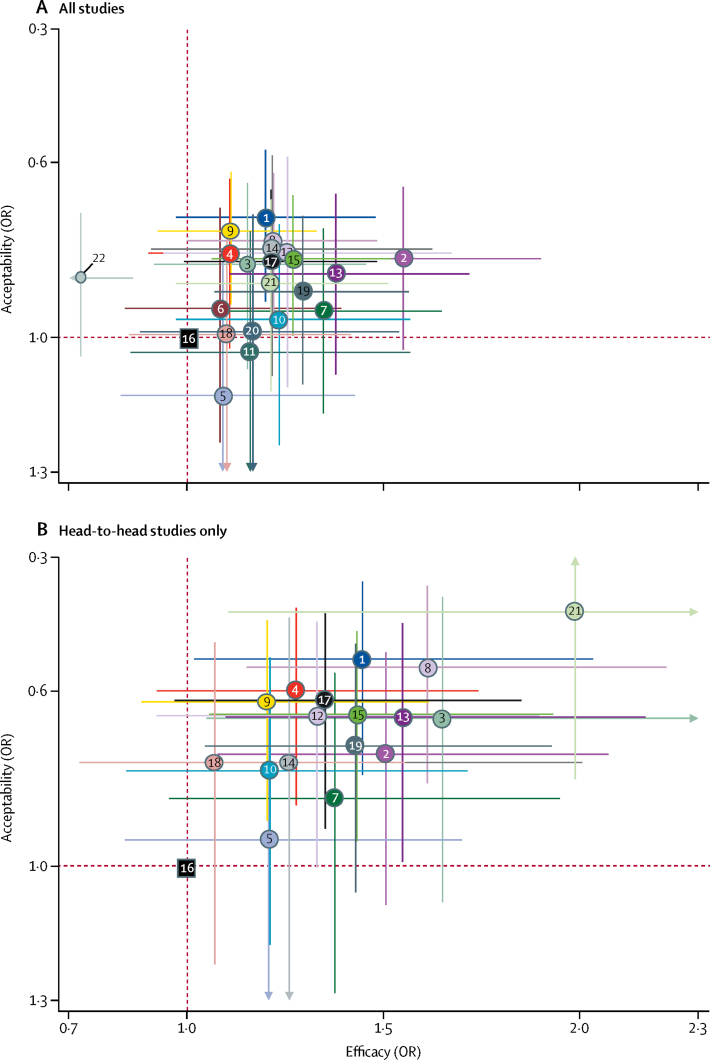
Two-dimensional graphs about efficacy and acceptability in all studies (A) and head-to-head (B) studies only Data are reported as ORs in comparison with reboxetine, which is the reference drug. Error bars are 95% CrIs. Individual drugs are represented by different coloured nodes. Desvenlafaxine, levomilnacipran, and vilazodone were not included in the head-to-head analysis because these three antidepressants had only placebo-controlled trials. ORs=odds ratios. 1=agomelatine. 2=amitriptyline. 3=bupropion. 4=citalopram. 5=clomipramine. 6=desvenlafaxine. 7=duloxetine. 8=escitalopram. 9=fluoxetine. 10=fluvoxamine. 11=levomilnacipran. 12=milnacipran. 13=mirtazapine. 14=nefazodone. 15=paroxetine. 16=reboxetine. 17=sertraline. 18=trazodone. 19=venlafaxine. 20=vilazodone. 21=vortioxetine. 22=placebo.

We also synthesised head-to-head studies separately to assess the differences between drugs. Figure 4 presents these data for the primary outcomes (194 studies with at least two active groups at licensed dose and comprised 34 196 patients). Agomelatine, amitriptyline, escitalopram, mirtazapine, paroxetine, venlafaxine, and vortioxetine were more effective than other antidepressants (ORs ranging between 1·19 and 1·96), whereas fluoxetine, fluvoxamine, reboxetine, and trazodone were among the least efficacious drugs (ORs ranging between 0·51 and 0·84). In terms of acceptability, agomelatine, citalopram, escitalopram, fluoxetine, sertraline, and vortioxetine were more tolerable than other antidepressants (ORs ranging between 0·43 and 0·77), whereas amitriptyline, clomipramine, duloxetine, fluvoxamine, reboxetine, trazodone, and venlafaxine were the antidepressants associated with the highest dropout rates (ORs ranging between 1·30 and 2·32). When all trials were considered, differences in ORs between antidepressants ranged from 1·15 to 1·55 for efficacy (appendix p 147) and from 0·64 to 0·83 for acceptability (appendix p 149), with wide CrIs on most of the comparative analyses. Figure 5 reports the two-dimensional graphs about efficacy and acceptability in all studies and head-to-head studies. Results for the secondary outcomes were in line with the findings for the primary outcomes (appendix pp 197–230). Within the head-to-head comparisons, when a treatment was the novel or experimental drug of comparison, it appeared to be significantly more effective than when that same treatment was the older or control drug of comparison (difference 1·18-times, 95% CrI 1·09–1·27). Adjusting for this novelty effect diminished the differences between antidepressants.

We incorporated the GRADE judgments in figure 4. The certainty of evidence for the relative treatment effects of efficacy and acceptability varied; it was moderate for most of the comparisons involving agomelatine, escitalopram, citalopram, and mirtazapine, and low to very low for most comparisons involving vortioxetine, nefazadone, clomipramine, bupropion, and amitriptyline (appendix pp 231–65). The appendix (pp 266–85) presents the ranking of treatments based on cumulative probability plots and SUCRAs.

In accordance with the review protocol, we also did a sensitivity analysis, including all the studies that used the drugs within the accepted doses (ie, doses recommended in some international clinical guidelines; appendix pp 133, 134) and the results did not change substantially (appendix p 187).

## Discussion

This study is based on 522 double-blind studies, which included 116 477 patients randomly assigned to 21 individual first-generation and second-generation antidepressant drugs or placebo. The project extends our previous work that had addressed 12 antidepressants with data for head-to-head comparisons.^9^ The present analysis is substantially more comprehensive because it includes 21 active treatments and placebo. The much larger evidence base (about 117 000 *vs* 26 000 patients), obtained through exhaustive search for published and unpublished information, allowed us to investigate additional important outcomes, such as remission, change in mood symptoms and dropouts due to side-effects, and a number of methodological issues, such as sponsorship, dosing schedule, study precision, and novelty effect.^16^

We found that all antidepressants included in the meta-analysis were more efficacious than placebo in adults with major depressive disorder and the summary effect sizes were mostly modest. Some antidepressants, such as escitalopram, mirtazapine, paroxetine, agomelatine, and sertraline had a relatively higher response and lower dropout rate than the other antidepressants. By contrast, reboxetine, trazodone, and fluvoxamine were associated with generally inferior efficacy and acceptability profiles compared with the other antidepressants, making them less favourable options. To make our results as relevant and robust as possible to inform clinical practice, we decided to focus on head-to-head studies and at the same time emphasise the certainty of the retrieved evidence. Our assessment overall found few differences between antidepressants when all data were considered, while there was more diversity in the range of efficacy and dropout patterns seen across the head-to-head comparisons than the meta-analysis of antidepressants versus placebo.

The present findings in adults contrast with the efficacy of antidepressants in children and adolescents, for which fluoxetine is probably the only antidepressant that might reduce depressive symptoms.^21^ This differential efficacy across age groups might reflect heterogeneous mechanisms and causes of depression,^22^ smaller number of studies in young people, or different methodological issues affecting adult and paediatric trials.^23^ The effect sizes were also smaller in more recent and larger placebo-controlled trials than in older and smaller ones, which might be an indicator of bias.

Estimated differences between drugs were smaller in placebo-controlled trials than in head-to-head studies. There are several potential explanations, as many factors have been associated with higher placebo response rates, such as randomisation ratio and the expectation of receiving an active treatment, the therapeutic setting, or the frequency of study visits.^24^ In our dataset, we found that response to the same antidepressant was on average smaller and dropouts more likely to occur in placebo controlled trials than in head-to-head studies. Moreover, for the same drug and the same probability of receiving placebo, larger all-cause dropout rates were associated with a lower response to treatment. The use of the last observation carried forward (LOCF) approach for imputing missing outcome data might have affected the estimates of treatment effect.^25^ Depressive symptoms tend to spontaneously improve over time and this phenomenon contributes to the high percentage of placebo responders in antidepressant trials.^26^ Patients randomly assigned to the active drug in a double-blind, placebo-controlled trial might leave studies earlier than in head-to-head studies because they might suspect they have been allocated to the placebo group than to the intervention group. Antidepressants usually take full effect only after weeks of treatment; therefore, participants who dropped out earlier tend to have poorer responses than those who remain on treatment, which are carried forward to the end of the trial by the LOCF analysis. The final result can be an underestimate of the true efficacy of the active drug.

Another possible explanation could be a bias in conduct, analysis, or reporting of head-to-head trials, driven by commercial interests.^27^ In our analyses, funding by industry was not associated with substantial differences in terms of response or dropout rates. However, non-industry funded trials were few and many trials did not report or disclose any funding. We also observed that drugs tended to show a better efficacy profile when they were novel and used as experimental treatments than when they had become old. This novelty effect might arise where a novel agent is perceived to be more effective and better tolerated; alternatively, selective analyses and outcome reporting bias might be more prominent when a treatment is first launched.^16^

Our literature search was as comprehensive as possible, including the largest amount of unpublished data to date, which are associated with less favourable effect sizes for antidepressants.^28^ Although it is possible that a certain amount of unpublished data could not be retrieved, our comparison-adjusted funnel plots did not suggest that small studies gave different results from larger studies either among placebo-controlled trials or head-to-head comparison trials (appendix pp 179–81, 225–27). The estimates of treatment effect from our study are in line with previous reviews on the same matter,^28^ but they are considerably more precise because of our larger quantity of data and resulting statistical power.

Our review has some limitations. According to the GRADE framework, the quality of many comparisons was assessed as low or very low for amitriptyline, bupropion, and venlafaxine, whereas it was often rated as moderate for agomelatine, escitalopram, and mirtazapine. We incorporated the certainty of evidence in the main results of our analysis to highlight the most robust findings for further use in clinical judgment. However, many trials did not report adequate information about randomisation and allocation concealment, which restricts the interpretation of these results. To increase the methodological rigour of the contributing evidence, we included only double-blind trials, which were generally very similar in design and conduct. The poor information in terms of risk of bias assessment might be a matter of reporting; however, we presented full details about the risk of bias of all included studies in the appendix (pp 115–32). We did not do a formal cost-effectiveness analysis. All of the most effective antidepressants are now off patent and available in generic form. Some of the antidepressants are included in the WHO Model List of Essential Medicines, which makes them available worldwide and ready to use also in developing countries.

We analysed only average treatment effects and were not able to investigate potentially important clinical and demographical modifiers of treatment response at the individual patient level (eg, age, sex, severity of symptoms, or duration of illness). Patients recruited in randomised trials tend to be highly selected and we also excluded patients with psychotic or treatment-resistant depression, which might limit the applicability of the results to these clinical subgroups, but it was intended as a methodological strength to assure transitivity in the network. We did not cover important clinical issues that might inform treatment decision making in routine clinical practice (eg, specific adverse events, withdrawal symptoms, or combination with non-pharmacological treatments). Additionally, because of the paucity of information reported in the original studies, we were not able to quantify some outcomes, such as global functioning. It should also be noted that some of the adverse effects of antidepressants occur over a prolonged period, meaning that positive results need to be taken with great caution, because the trials in this network meta-analysis were of short duration. The current report summarises evidence of differences between antidepressants when prescribed as an initial treatment. Given the modest effect sizes, non-response to antidepressants will occur. Our information unfortunately cannot guide next-step choices after failure of such a first step (ie, they do not apply to treatment-resistant depression), for which well performed trials are scarce.^29^

Using the data made available on the websites of the US Food and Drug Administration and European Medicines Agency, on the international trial registries, and from contacting study authors and pharmaceutical companies, we managed to incorporate in the analysis a considerable amount of unpublished data for some drugs—namely, agomelatine, escitalopram, paroxetine, reboxetine, sertraline, venlafaxine, vilazodone, and vortioxetine—but not for all the antidepressants included in the network meta-analysis. This limitation in the primary trials might affect the validity of the findings for some antidepressants, but the incorporation of both direct and indirect comparisons might have contributed to reduce the potential risk of bias.^30^ We did our best to retrieve all unpublished data and contacted study authors for supplemental material, but we are aware that a substantial amount of information is still not available to the public. There are online archives where trials are prospectively registered; however, they collect reliable information only about the most recent studies and we cannot rule out the possibility that some studies are absent or the same study has been counted twice in our analyses. It is not uncommon for the same study to go by different names in different publications, which complicates the process of data synthesis.^31^ By making the dataset fully and freely available, we welcome any information that might help clarify any mistakes in our dataset.

Notwithstanding these limitations, the findings from this network meta-analysis represent the most comprehensive currently available evidence base to guide the initial choice about pharmacological treatment for acute major depressive disorder in adults. All statements comparing the merits of one antidepressant with another must be tempered by the potential limitations of the methodology,^32^ the complexity of specific patient populations, and the uncertainties that might result from choice of dose or treatment setting. We hope that these results will assist in shared decision making between patients, carers, and their clinicians.
